# Targeting Human-Cytomegalovirus-Infected Cells by Redirecting T Cells Using an Anti-CD3/Anti-Glycoprotein B Bispecific Antibody

**DOI:** 10.1128/AAC.01719-17

**Published:** 2017-12-21

**Authors:** Weixu Meng, Aimin Tang, Xiaohua Ye, Xun Gui, Leike Li, Xuejun Fan, Robbie D. Schultz, Daniel C. Freed, Sha Ha, Dai Wang, Ningyan Zhang, Tong-Ming Fu, Zhiqiang An

**Affiliations:** a Texas Therapeutics Institute, Brown Foundation Institute of Molecular Medicine, University of Texas Health Science Center at Houston, Houston, Texas, USA; b Merck Research Laboratories, Merck & Co., Inc., Kenilworth, New Jersey, USA

**Keywords:** HCMV, bispecific antibody, T cell immunity, therapeutic agent, T cell activation

## Abstract

The host immune response to human cytomegalovirus (HCMV) is effective against HCMV reactivation from latency, though not sufficient to clear the virus. T cells are primarily responsible for the control of viral reactivation. When the host immune system is compromised, as in transplant recipients with immunosuppression, HCMV reactivation and progressive infection can cause serious morbidity and mortality. Adoptive T cell therapy is effective for the control of HCMV infection in transplant recipients. However, it is a highly personalized therapeutic regimen and is difficult to implement in routine clinical practice. In this study, we explored a bispecific-antibody strategy to direct non-HCMV-specific T cells to recognize and exert effector functions against HCMV-infected cells. Using a knobs-into-holes strategy, we constructed a bispecific antibody in which one arm is specific for CD3 and can trigger T cell activation, while the other arm, specific for HCMV glycoprotein B (gB), recognizes and marks HCMV-infected cells based on the expression of viral gB on their surfaces. We showed that this bispecific antibody was able to redirect T cells with specificity for HCMV-infected cells *in vitro*. In the presence of HCMV infection, the engineered antibody was able to activate T cells with no HCMV specificity for cytokine production, proliferation, and the expression of phenotype markers unique to T cell activation. These results suggested the potential of engineered bispecific antibodies, such as the construct described here, as prophylactic or therapeutic agents against HCMV reactivation and infection.

## INTRODUCTION

Human cytomegalovirus (HCMV) is an important pathogen, ubiquitous in human populations, with >50% prevalence in adults worldwide. Primary HCMV infection rarely causes serious disease in healthy subjects, and the infection is quickly controlled by the host immune system. HCMV establishes latent infection after the resolution of the primary infection, and viral reactivation is effectively controlled by the HCMV-specific host immunity. However, when host immune systems are compromised, as they are in solid-organ or hematopoietic stem cell transplant recipients under immunosuppression, HCMV can be reactivated, leading to widespread viral replication and dissemination to multiple organs. Reactivation and infection could be life-threatening if not managed actively (1, 2).

Current strategies for preventing HCMV reactivation and infection in transplant recipients include (i) universal prophylaxis, which involves antiviral medications for approximately 100 to 200 days posttransplantation, and (ii) preemptive prophylaxis, in which antiviral therapy is initiated for patients whose viral loads reach a certain threshold indicative of active viral infection (3, 4). Although both strategies have been successful in reducing HCMV-related morbidity and mortality, the side effects of the antiviral drugs and the emergence of drug-resistant HCMV mutants are constant concerns in the clinic (5–8). Another approach is adoptive immunotherapy, in which *in vitro*-cultured autologous HCMV-specific T cells have been adoptively transferred to patients who have developed drug-resistant HCMV infection and disease. This approach is beneficial for long-term reconstitution of protective antiviral immunity, with disease-free survival and no allograft rejection for patients (9). However, due to genetic diversity, adoptive T cell therapy must be tailored to each individual. Further, the success of adoptive T cell therapy depends on successful recovery of autologous anti-HCMV T cells and their expansion in culture. This therapeutic regimen is technically sophisticated, labor-intensive, and difficult to implement in routine clinical practice.

To overcome the difficulties associated with adoptive T cell therapies, bispecific T-cell-engaging (BiTE) antibodies have been designed and developed. BiTE antibodies are able to bypass the requirement for T cell activation through T cell receptor (TCR) engagement with autologous antigens presented by major histocompatibility complex class I (MHC-I) molecules; instead, they activate T cells through generic CD3 interaction. The activated T cells are redirected to the target sites through the recognition of a virus- or tumor-specific antigen by BiTE antibodies (10). This concept has been tested for HCMV-infected cells by use of chemically conjugated bispecific antibodies (BsAbs). A murine IgG2a anti-CD3 monoclonal antibody (MAb), OKT3, was chemically linked to CytoGam, an HCMV hyperimmune immunoglobulin (HCMV HIG) (11). The chemically conjugated antibodies were able to mediate specific cytotoxicity at HCMV-infected target cells (11). However, the BsAbs from this type of chemical conjugation, especially with HCMV HIG as polyclonal antibodies, are complex in pharmaceutical composition and difficult to control for consistency in manufacturing. For this reason, a single bispecific molecule is preferred. One of the well-developed BsAb platforms is the “knobs-into-holes” (KIH) strategy (12). Knobs are created by replacing the residues with small side chains at the interface between CH3 domains of the immunoglobulin (Ig) constant region with those of larger side chains, whereas holes are constructed by replacing the residues with large side chains with those of smaller side chains. The noncovalent interactions, along with disulfide bridges in the hinge region, drive assembly toward Ig heterodimer formation and yield >92% heterodimers (12–14).

In this study, we engineered a KIH BsAb with one arm targeting CD3 for the activation of T cells and the other arm specific to the major viral glycoprotein gB. The T cell activation arm was derived from MAb OKT3, specific to the CD3ε chain (15), and the viral specificity arm was derived from a humanized rabbit MAb, hu272.7, with high affinity for HCMV gB but no virus-neutralizing activity (16). We detected gB on the surfaces of HCMV-infected cells. The high affinity and specificity of hu272.7 for gB ensures that the BsAb molecule will react only to HCMV-infected cells. We demonstrated here that this bispecific antibody was able to mediate T-cell-specific activation and effector functions for HCMV-infected target cells. The results validated the design concept and provided *in vitro* evidence that HCMV-infected cells can be targeted functionally by the anti-CD3/anti-gB bispecific antibody in the presence of human T cells regardless of the donor's genetic background. The results further suggested that this bispecific construct warrants further evaluations in the clinic as a prophylaxis and an alternative to the standard chemical antivirals for the prevention of HCMV infection and of reactivation posttransplantation.

## RESULTS

### Humanization of an anti-gB antibody.

**FIG 1:**
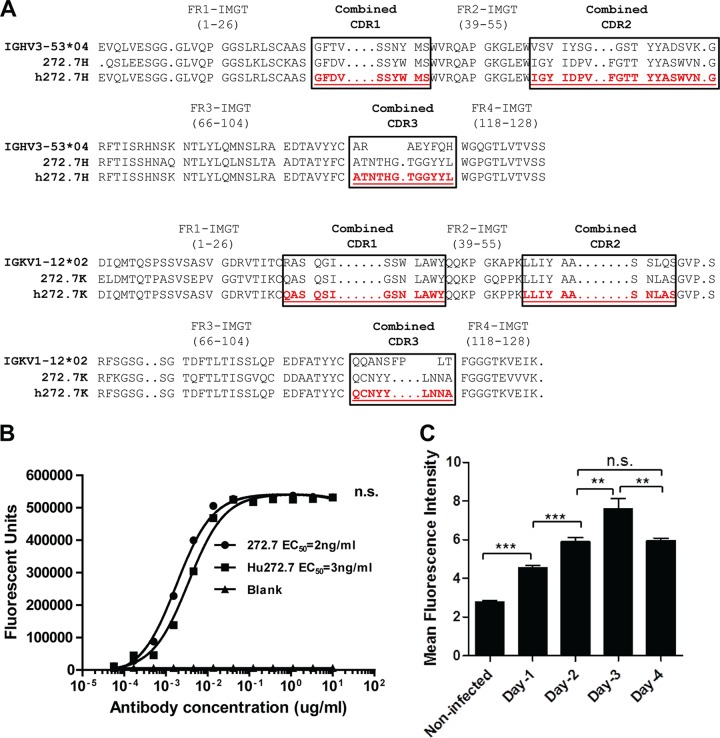
Humanization of a rabbit HCMV gB-specific antibody and detection of gB expression on the surfaces of HCMV-infected cells. (A) Sequence alignment of the closest human germ lines (IGHV3-53*04), rabbit antibody 272.7, and the humanized antibody (hu272.7). The combined CDRs determined are boxed. Antibody humanization was performed by CDR grafting. (B) The humanized antibody maintained affinity and specificity for gB. The rabbit 272.7 and hu272.7 antibodies in titration were tested for binding to gB protein by ELISA. EC_50_s were deduced from four-parameter curve fitting. The statistical significance of differences between the rabbit 272.7 and hu272.7 antibodies was analyzed by two-way ANOVA. n.s., not significant (*P* > 0.05). (C) Detection of gB expression on the surfaces of HCMV-infected ARPE-19 cells by a flow cytometry assay. The mean fluorescence intensities ± SD of gB-specific signals from triplicate samples are shown. The data are representative results from two independent experiments. Statistical significance was determined by the unpaired two-tailed *t* test. **, *P* < 0.01; ***, *P* < 0.001.

To construct an HCMV-specific and T-cell-engaging bispecific antibody (BsAb), we selected a high-affinity anti-HCMV gB antibody, hu272.7 (16), to confer specificity for HCMV. Antibody hu272.7 is a humanized form of the anti-gB rabbit MAb (16). Humanization was achieved by complementarity-determining region (CDR) grafting, and the substitution of each amino acid in the framework region is shown in Fig. 1A. The design was performed via grafting combined Kabat/IMGT/Paratome complementarity-determining regions (17, 18). Antibody hu272.7 maintained the affinity of the original rabbit antibody, 272.7, as evidenced by the fact that the effective concentration of IgG to reach 50% of the binding signal (EC_50_) of hu272.7, 3 ng/ml, was comparable to the EC_50_ for the parental antibody 272.7, 2 ng/ml (Fig. 1B).

For the bispecific-antibody strategy to work, it is essential to detect HCMV gB proteins on the surfaces of infected host cells. A flow cytometry assay was used to determine whether hu272.7 could detect gB on the surfaces of infected cells. HCMV-infected (multiplicity of infection [MOI], 10) ARPE-19 cells were stained with hu272.7 at days 1, 2, 3, and 4 postinfection. As shown in Fig. 1C, HCMV-infected ARPE-19 cells showed higher gB-specific signals than noninfected cells, and the intensities of the signals increased in a time-dependent manner. The mean fluorescence intensity of the gB-specific signal in infected cells at day 1 was significantly higher than that in noninfected cells. The gB-specific signal increased significantly daily until day 3 and began to drop at day 4 postinfection. This result demonstrated that hu272.7 can positively detect gB expression on HCMV-infected cells.

### Design of a bispecific antibody to redirect T cells to HCMV infection.

**FIG 2:**
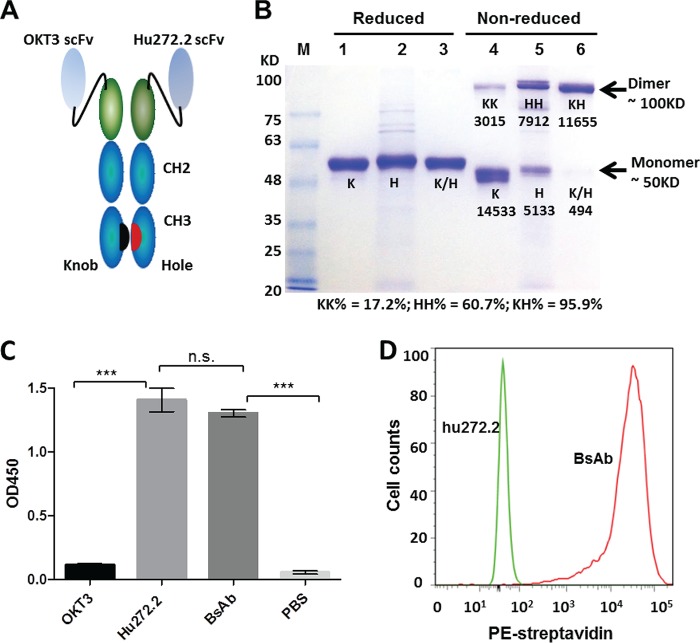
Design and characterization of the bispecific antibody (BsAb). (A) Schematic depiction of the bispecific antibody with gB and CD3 specificities. (Gly_4_Ser)_3_ linkers were constructed between the V_H_ and V_L_ domains of their respective scFvs. (B) Bispecific-antibody expression in HEK293F cells. Single-chain controls and the bispecific antibody were expressed by transfection of the scFv-K-Fc or scFv-H-Fc construct, or both, and were affinity purified with protein A. The constructs were then evaluated by SDS-PAGE under either reducing conditions (lanes 1 to 3) or nonreducing conditions (lanes 4 to 6). Lanes 1 and 4, single-chain hu272.7 scFv-K proteins; lanes 2 and 5, OKT3 scFv-H; lanes 3 and 6, the bispecific antibody. K, knob monomer; H, hole monomer; K/H, knob or hole mixed monomer; KK, knob homodimer; HH, hole homodimer; KH, knob-into-hole heterodimer. The densitometry values of the bands were analyzed by ImageJ software and are shown below the bands in lanes 4 to 6. The percentages of dimers were calculated and are shown below the gel. (C) Specificity of the BsAb for immobilized gB as determined by ELISA. Statistical significance was determined by two-way ANOVA. (D) Testing of the specificities of the BsAb (red) and the hu272.7 antibody (green) for T lymphocytes by flow cytometry. The binding signal is detected by a PE-conjugated anti-human Fc antibody.

Antibody hu272.7 was used as one arm of the bispecific-antibody design. The functional arm for activating T cells was from anti-human CD3 MAb OKT3 (19). Both arms were designed as single-chain variable fragments (scFvs) (20). Our bispecific-antibody vectors were designed based on the knobs-into-holes concept, which has demonstrated effective dimerization of two different IgG heavy chains between Fc regions (14, 21). The constructs, as shown in Fig. 2A, were composed of two scFvs, one targeting gB and one targeting CD3, directly fused to their respective hole and knob Fc regions (Fig. 2A).

To verify the design, the scFv-Fc KIH constructs were expressed by transient transfection in HEK293 cells. Single constructs, designated K (knob) or H (hole), were also transfected as controls. The bispecific antibody and its single-chain control antibodies were purified using protein A affinity chromatography and were tested for purity by SDS-PAGE under both reducing and nonreducing conditions (Fig. 2B). Under reducing conditions, all single-chain constructs showed a protein band of the expected size, around 50 kDa. Under nonreducing conditions, the single-chain control arm of the scFv-K-Fc construct showed a tendency to form monomers, and there was a faint band representing a KK dimer, accounting for about 17.2% of the population. In contrast, about 60.7% of proteins from the single-chain control arm of scFv-H-Fc were HH dimers. More importantly, cotransfection of scFv-K-Fc and scFv-H-Fc yielded correctly paired KH dimers with 95.9% pairing efficiency as assessed by densitometry (Fig. 2B).

Enzyme-linked immunosorbent assays (ELISA) and flow cytometry were used to confirm the dual specificity of the BsAb against recombinant HCMV gB and against CD3 on T cells. The parental anti-gB antibody hu272.7 and the bispecific antibody exhibited comparable reactivities to recombinant gB immobilized on a solid surface, with optical density (OD) readings around 1.4 and 1.3, respectively; as expected, the anti-CD3 antibody OKT3 did not react to gB (Fig. 2C). Since OKT3 recognizes a conformational epitope that could not be presented with recombinant CD3ε monomeric protein (data not shown), we evaluated the binding of the bispecific antibody to human T cells with a native CD3 polymeric membrane protein complex by use of a flow cytometry assay. As shown in Fig. 2D, the bispecific antibody, but not the anti-gB antibody hu272.7, showed binding to purified T cells.

### Characterizations of the bispecific antibody for its dual specificity and function.

**FIG 3:**
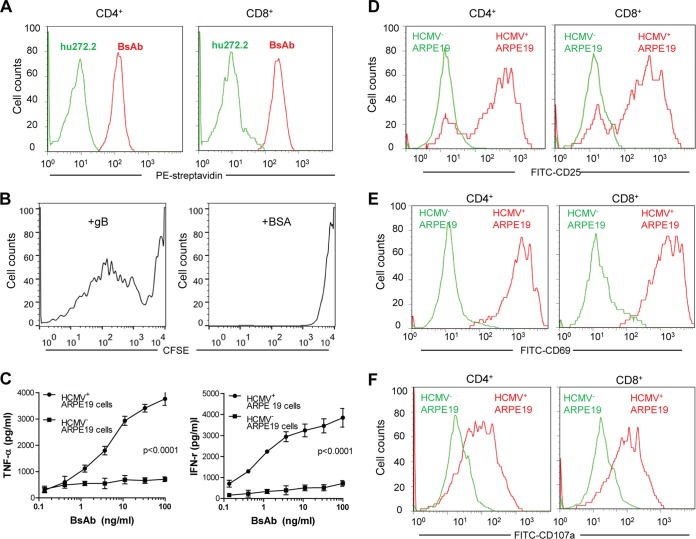
The bispecific antibody activates T cells, as shown by the proliferation and secretion of Th1-type cytokines. (A) Dual specificity of the bispecific antibody. Biotinylated gB protein was incubated with CD4 or CD8 T cells pretreated with the bispecific (red) or hu272.7 (green) antibody. PE-conjugated streptavidin was used to detect the binding signal as an indication of dual specificity. (B) Antigen-specific proliferation of T cells mediated by the bispecific antibody. T cells were prestained with CFSE and were then incubated with the bispecific antibody in culture plates coated with recombinant gB protein (left) or BSA (right) for 2 days. The proliferation of T cells in response to the antibody treatment was then determined by measuring the CFSE signal by flow cytometry. (C) Activation of T cells as shown by cytokine production. T cells were incubated with HCMV-infected (circles) or uninfected (squares) ARPE-19 cells in the presence of the bispecific antibody in titration. The supernatants were collected 2 days later for the measurement of TNF-α and IFN-γ production. Statistical significance was determined by two-way ANOVA. (D to F) Two days after the incubation of HCMV-infected (red) or uninfected (green) ARPE-19 cells with the BsAb, T cells were analyzed by flow cytometry for activation markers CD25 (D) and CD69 (E) and for degranulation marker CD107a (F).

To confirm that the BsAb can react to both T cells and the gB protein simultaneously, we performed flow cytometry and assessed the dual binding capacity of the BsAb. The assay is performed by adding the bispecific antibody and biotinylated recombinant gB protein sequentially to purified human CD4 or CD8 T cells. Dual binding was detected using phycoerythrin (PE)-labeled streptavidin, which can label only the biotinylated gB. The flow cytometry data showed that the bispecific antibody can react to T cells and gB protein through its two respective arms, while the parent antibody yielded no binding signal in the dual binding assay (Fig. 3A). These results demonstrated the dual specificity of the BsAb for both human CD4 and CD8 T cells as well as recombinant gB protein and suggested its potential to redirect T cells to HCMV-infected cells, which are marked by viral gB proteins, as we have demonstrated (Fig. 1C).

The ability of the BsAb to activate T cells *in vitro* was tested in several different assays. First, we evaluated the ability of the bispecific antibody to induce antigen-specific proliferation of T cells, as measured by dilution of the fluorescent signal of the in-cell dye carboxyfluorescein diacetate succinimidyl ester (CFSE). Recombinant gB or bovine serum albumin (BSA) was immobilized on a solid surface in culture plates as the cognate antigen source. Then soluble BsAb was incubated with CFSE-labeled T cells. Proliferation, as revealed by CFSE dilution in lymphocytes, was measured by flow cytometry. As shown in Fig. 3B, soluble BsAb in the presence of immobilized gB led to multiple populations of diluted CSFE signals, indicating several rounds of T cell proliferation, while no such proliferation pattern was observed with BSA as the reactive antigen. Further analysis revealed that 71.1% of T cells were activated when incubated with gB protein, while only 2.6% of T cells were activated when incubated with the control antigen BSA. Thus, the interaction between the BsAb and the gB target is essential for triggering T cell proliferation *in vitro*.

Second, effective antivirus immune responses are known to require the secretion of inflammatory cytokines, in particular tumor necrosis factor alpha (TNF-α) and gamma interferon (IFN-γ) (22). To test whether activation of T cells by the BsAb can lead to cytokine production, we quantified TNF-α and IFN-γ secretion in peripheral blood mononuclear cells (PBMCs) cultured with the bispecific antibody and in HCMV-infected or uninfected ARPE-19 cells. Culture supernatants from control cells with no HCMV infection had minimal levels of these two cytokines—10 to 200 pg/ml—even when the bispecific antibody concentration reached 0.1 μg/ml (Fig. 3C). In contrast, when incubated with HCMV-infected ARPE-19 cells, the BsAb elicited significantly high levels of TNF-α and IFN-γ production; the concentrations of both TNF-α and IFN-γ reached about 4,000 pg/ml (Fig. 3C). The production of these two cytokines in a dose-response fashion indicated that the BsAb was capable of recognizing gB on the surfaces of infected cells and, more importantly, that it was able to engage and activate T lymphocytes.

Lastly, we evaluated the phenotypic markers for T cell activation after engagement of the bispecific antibody with HCMV-infected ARPE-19 cells. CD4 and CD8 T cells showed upregulated surface expression of the activation markers CD25 and CD69 and the degranulation marker CD107a (Fig. 3D through F). Importantly, these changes in phenotypic markers required both the presence of the BsAb in culture and HCMV infection, since elevated expression of CD25, CD69, and the degranulation marker CD107a was detected only in the presence of HCMV-infected ARPE-19 cells, not in uninfected ARPE-19 cells (Fig. 3D through F). These data collectively demonstrated that the BsAb is functionally active in engaging virus-specific antigen, either presented as a recombinant protein or expressed on the surfaces of HCMV-infected cells, and is capable of triggering T cell activation through its specificity for CD3.

### Redirecting T cells to HCMV-infected ARPE-19 cells.

**FIG 4:**
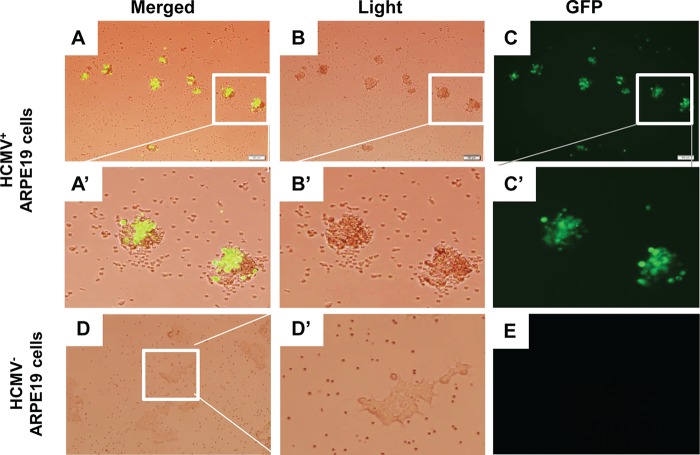
Light microscopy of T cells incubated with HCMV-infected or uninfected ARPE-19 cells in the presence of the bispecific antibody. T cells and the bispecific antibody were added to a 6-well plate that was seeded with HCMV-infected (as indicated by GFP) or uninfected ARPE-19 cells. Fluorescence microscopy images were taken 48 h later. (A′ and B′) T cells migrated toward HCMV-infected ARPE-19 cells. (D′) T cells were evenly dispersed in the wells with uninfected ARPE-19 cells. Magnification, ×10 for panels A, B, C, D, and E; ×40 for panels A′, B′, C′, and D′.

To further confirm the specificity of the BsAb, we utilized microscopy to visualize T cells and HCMV-infected ARPE-19 cells. We observed the specific colocalization of T cells with infected cells, as shown in the merged image in the presence of the BsAb (Fig. 4A). The enlarged images in Fig. 4 (Fig. 4A′ and B′) contain two HCMV-infected cells (marked by green fluorescent protein [GFP] expression [Fig. 4C′]) surrounded by lymphocytes. In contrast, the T cells appeared to be distributed evenly in the presence of uninfected ARPE-19 cells, without obvious clustering (Fig. 4D and D′). This result provided further evidence that T cells were activated and redirected to HCMV-infected ARPE-19 cells by the bispecific antibody through the interactions with gB protein and CD3ε on target cells and T cells, respectively.

### T-cell-mediated cytotoxicity in ARPE-19 cells.

**FIG 5:**
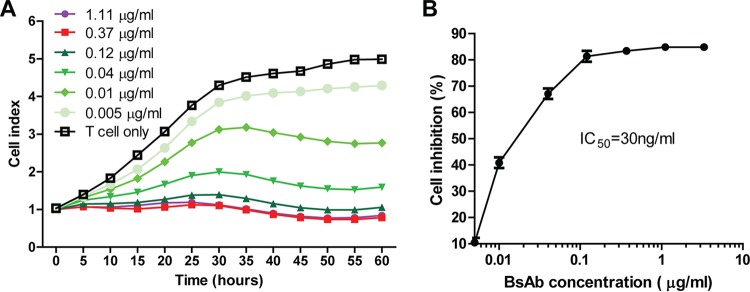
The bispecific antibody mediated T-cell-dependent inhibition of cell growth. (A) Bispecific antibodies in titration were incubated with T cells and HCMV-infected ARPE-19 cells. The electric impedance was monitored in real time for 60 h in order to map the cell growth curve, and the cell index curve was normalized to the growth of the control (HCMV-infected ARPE-19 cells, with no T cells). (B) The percentage of inhibition of the HCMV-infected cell varies proportionally with the antibody concentration. The percentage of T-cell-dependent inhibition of cell growth in response to the antibody was calculated using the following formula: [(cell index with T cells only) − (cell index of T-cell-plus-antibody treatment)]/(cell index with T cells only) × 100%.

We next sought to test the ability of the BsAb to elicit antigen-specific viral inhibition *in vitro*. Using the xCELLigence instrument, we monitored the viability of HCMV-infected ARPE-19 cells in the presence of human T cells as effector cells. We showed that the viability of ARPE-19 cells was not affected if the BsAb was absent from the culture, since the cell growth index increased steadily for 60 h. However, the BsAb-mediated redirection of T cells was inhibitory to the growth of HCMV-infected ARPE-19 cells, and this inhibition was demonstrated in a dose-response pattern from 0.005 to 1.11 μg/ml. The time course study showed that this efficacy against viral infection started to reach a plateau around 30 h after the addition of the antibody (Fig. 5A). The percentage of cell inhibition was calculated based on the cell growth index at the 30-h time point (Fig. 5B). The cell growth inhibition effect seemed to plateau around 0.37 μg/ml, and the 50% inhibitory concentration (IC_50_) was calculated at ∼0.03 μg/ml. This result provided further evidence that the BsAb can mediate the inhibition of HCMV-infected ARPE-19 cells in a dose-dependent manner.

## DISCUSSION

Latent activation of HCMV infection can be lethal to immunocompromised patients, such as transplant recipients under immunosuppressive regimens. It is feasible to eliminate the cell-free virus with neutralizing antibodies. This concept is supported by multiple reports on the treatment of viremia by HCMV-neutralizing antibodies (23–26). For prophylactic regimens against latent HCMV, it is crucial to inhibit HCMV viral reactivation under conditions under which the viral antigens are not visible to antibodies. In this study, we demonstrated the feasibility of a bispecific antibody capable of inhibiting viral infection through activation of nearby T cells. The BsAb as designed (i) could maintain dual specificity for the viral antigen and CD3, (ii) was able to trigger T cell activation with engagement of the viral target, and (iii) inhibited the growth of HCMV-infected cells. The results validated the utility of the bispecific antibody as designed for a prophylactic or therapeutic agent against HCMV. The concept of BiTE was established with the recent approval of blinatumomab for oncology targets (27). The technology has also been used for the treatment of chronic virus infection; an anti-CD3/anti-gp120 bispecific antibody has been shown to be able to kill host cells infected with HIV (28). However, there are many design details that would be critical for the performance as well as the evaluation of the bispecific antibody for HCMV infection.

First, we selected the virus-specific arm with an antibody targeting gB. We hypothesized that since gB is one of the most abundant HCMV envelope proteins (29), even though it is a late viral protein, it would be sufficient for the recognition of viral infection by the expression of gB on the cell surface even during the early phase of infection. Indeed, we were able to detect gB proteins on the surfaces of HCMV-infected cells (Fig. 1C). We demonstrated the specificity of our BsAb for virus-infected ARPE-19 cells within 2 days of viral infection for T cell activation (Fig. 3D) or T-cell-mediated inhibition of viral infection (Fig. 4). Second, we chose a rabbit MAb, 272.7, with high affinity to gB but with no neutralizing activity *in vitro*. This choice simplified the interpretation of the result, since the antiviral effect of the BsAb was unlikely due to viral neutralization. Third, OKT3, the first commercial antibody that can bind CD3ε and activate T cells, was chosen as the functional arm of the bispecific antibody, since it has been used in bispecific-antibody designs for cancer and viral therapies (30–33).

The efficiency of KH heterodimer formation and the configuration of the BsAb were also important considerations for the design of bispecific antibodies. Mispairing of the heavy chain and light chain would create unwanted homodimers. In our case, one of the inherent safety issues was the contamination of homodimers with CD3 specificity; such contaminated homodimers can activate T cells without any viral specificity, thus posing safety risks. Thus, the “knobs into holes” design, which has been reported to yield >92% heterodimers in the product (13), was selected. By engrafting single-chain Fvs of OKT3 and 272.7 onto the Fc-K and Fc-H constructs, respectively, we demonstrated robust production of the bispecific antibody by cotransfection of two DNA constructs. Furthermore, the BsAb by design would retain the full Fc functions with a half-life expected of an IgG. More importantly, since the OKT3 scFv was engrafted on Fc-K, not Fc-H, the Fc-K was unlikely to form the KK homodimer due to its bulk side-chain modification in the Fc regions. Our data showed that single-construct transfection of scFv-K-Fc could yield only 17.2% homodimer, in contrast to 60.7% for scFv-H-Fc (Fig. 2B). The pairing efficiency for the bispecific antibody, as assessed by densitometry, was >95%. It should be noted that, due to similar molecular sizes, it would be difficult to determine the levels of KK dimer contamination within the heterodimer. A more precise method, such as mass spectrometry, may be needed to control this contaminated portion prior to clinical evaluation.

The dual specificity of our BsAb was extensively verified, either by biochemical experiments or indirectly, by functional evaluations. The expression of gB on the surfaces of HCMV-infected cells is expected to be low in the early phase of viral infection, but we were able to detect gB proteins on the surfaces of HCMV-infected ARPE-19 cells by flow cytometry 1 day postinfection. The levels of gB proteins on the surfaces of HCMV-infected ARPE-19 cells gradually increased 1, 2, and 3 days postinfection, and the expression reached a plateau on day 3 postinfection. Interestingly, the BsAb could mediate T cell migration and activation in the presence of HCMV-infected cells. First, HCMV infection was a trigger for T cell aggregation (Fig. 4). Second, IFN-γ production and TNF-α production were dependent on HCMV infection (Fig. 3C). Third, the phenotypic markers associated with T cell activation and degranulation, such as CD25, CD69, and CD107a, were detected in the presence of HCMV-infected cells (Fig. 3D, E, F). These results suggested that the level of gB expression on the membranes of HCMV-infected cells, although low and not sufficient for detection by flow cytometry in the early phase of infection, was still above the threshold for recognition by the BsAb, which, in turn, triggered T cell activation. The preexisting anti-gB antibodies in patients might pose a concern for clinical application of the BsAb by competing for the binding of gB on infected cells. This issue needs to be evaluated further in clinical settings.

CD3 is a common surface marker for all T cells and an extremely potent trigger for T cell activation. Thus, target-specific T cell activation has to be demonstrated for any bispecific antibodies aiming to redirect T cells. In our case, the bispecific antibody, even at a high concentration of 0.1 μg/ml, was not able to activate T cells, as measured by IFN-γ and TNF-α cytokine production, if ARPE-19 cells were not infected (Fig. 3C). Microscopy images also showed enriched migration of T cells to ARPE-19 cells only when infection was indicated (Fig. 4). Taken together, these results demonstrate that the BsAb can recruit and activate T cells with specificity for HCMV-infected cells.

In summary, we validated the concept of a bispecific antibody that can redirect T cells to HCMV-infected cells. The bispecific antibody based on the knobs-in-holes design can be produced with high pairing efficiency. The data presented in this study demonstrated the specificity of the bispecific antibody for HCMV-infected cells and its capacity to activate T cells for proliferation, cytokine production, and cytotoxicity for virus-infected cells. These results suggest the potential of bispecific antibodies such as the molecule described here as prophylactic or therapeutic agents against HCMV reactivation posttransplantation.

## MATERIALS AND METHODS

### Cell lines, PBMC and T cell isolation, virus, and reagents.

The ARPE-19 human retinal pigment epithelial cell line was purchased from the American Type Culture Collection (ATCC, Manassas, VA). Cells were cultured as recommended by the supplier. Peripheral blood mononuclear cells (PBMCs) were isolated from enriched lymphocyte preparations (buffy coats) obtained from a local blood bank by density gradient separation using Accuspin tubes (Sigma-Aldrich, St. Louis, MO) according to the manufacturer's instructions. Briefly, Accuspin tubes were centrifuged at 800 × *g* for 30 s at room temperature; then 10 ml Hanks buffer followed by 20 ml blood was added to the upper chamber of each tube. The tubes were then centrifuged at 1,000 × *g* for 10 min at room temperature. After centrifugation, the plasma layer was aspirated with a Pasteur pipette to within 0.5 cm of the opaque interface containing the mononuclear cells. The opaque interface above the frit was transferred with a Pasteur pipette to a clean conical centrifuge tube. The cells were then washed by adding 10 ml RPMI 1640 medium containing 20% fetal bovine serum (FBS) and were centrifuged at 300 × *g* for 10 min at room temperature. After the supernatant was discarded, cells were resuspended and were washed twice in 5 ml RPMI 1640 medium containing 20% FBS. PBMCs were prepared the same day that the buffy coats were received. The serum of the buffy coat was further tested via ELISA using inactivated revertant HCMV particles as the coating antigen. The result showed that the donor was HCMV seronegative.

T cells were enriched from PBMCs by use of a RapidSpheres T cell enrichment kit (Stemcell Technologies, Vancouver, BC, Canada) according to the manufacturer's instructions. Briefly, a 50-μl/ml isolation cocktail was added to a 5-ml polystyrene round-bottom tube containing 2 ml PBMCs at a density of 5 × 10^7^/ml and was incubated at room temperature for 5 min. RapidSpheres were vortexed for 30 s and were added to the incubated cells at 50 μl/ml of sample. A 4-ml volume of isolation medium was added to the sample and was mixed by gently pipetting up and down 2 to 3 times. The tube was transferred to the separation magnet and was incubated at room temperature for 3 min. Finally, the enriched T cell suspension was poured off into a new tube.

Recombinant HCMV gB protein (Sino Biologicals, Beijing, China) was based on the sequence of the Towne strain with its furin cleavage site mutated and the transmembrane region deleted (34). The parental virus, HCMV AD169 (ATCC, Manassas, VA), was propagated in MRC-5 cells (35). The AD169 pentameric gH complex revertant virus (36) and the revertant virus with GFP marker have been described previously (37).

### Construction of KIH bispecific antibodies.

To facilitate Fc heterodimer formation, we used a strategy developed by Carter and others (13, 14, 21) to introduce a set of “knobs-into-holes” (KIH) mutations into the CH3 domain of the Fc region. We first modified a full-length IgG expression vector, which contains an IgG1 constant region, by deleting the CH1 fragment to create an IgG-Fc backbone. The Fc KIH constructs were then designed based on the IgG-Fc backbone. For the Fc-knob (Fc-K) construct, one mutation was introduced into the CH3 domains as described previously (21) (T366W, designated “knob”) using a site-directed mutagenesis kit (Invitrogen, Carlsbad, CA). The primers for mutagenesis were T366W-F (5′-AACCAGGTGAGCCTGTGGTGCCTGGTGAAGGGC-3′) and T366W-R (5′-GCCCTTCACCAGGCACCACAGGCTCACCTGGTT). For the Fc-hole construct (Fc-H), three mutations were introduced into the CH3 domain as described previously (21) (T366S, L368A, and Y407V, designated “hole”) using a site-directed mutagenesis kit (Invitrogen, Carlsbad, CA). The primers for the mutagenesis were T366S, L368A-F (5′-AACCAGGTGAGCCTGTCCTGCGCCGTGAAGGGCTTCTAC-3′), T366S, L368A-R (5′-GTAGAAGCCCTTCACGGCGCAGGACAGGCTCACCTGGTT-3′), Y407V-F (5′-GGCTCCTTCTTCCTGGTTAGCAAGCTGACAGTG-3′), and Y407V-R (5′-CACTGTCAGCTTGCTAACCAGGAAGAAGGAGCC-3′).

To circumvent light-chain mispairing, we constructed a single-chain variable fragment (scFv), linking the heavy and light chains of the same antibody by a (Gly_4_Ser)_3_ linker. The scFv of either the anti-CD3 antibody or the anti-gB protein antibody was then cloned into the IgG-Fc-knob or IgG-Fc-hole vector, respectively. The antibody against the HCMV gB protein (272.7) was initially produced from a rabbit hybridoma as reported previously (16). The genes encoding the heavy and light chains of the antibody were cloned into an IgG1 vector as reported previously (16), and the antibody was humanized to yield antibody hu272.7. The humanization was achieved by CDR grafting. We first performed alignment for antibody 272.7 using NCBI/IGBLAST (https://www.ncbi.nlm.nih.gov/igblast/) to identify the most similar human germ line antibody. Then we defined the three CDRs of 272.7 based on three different nomenclature systems: IMGT, Kabat/Chothia, and Paratome. The amino acids in the framework regions of the rabbit antibody that differed from the aligned human germ line antibody were replaced with corresponding human residues. The humanized antibody sequence was synthesized as hu272.7 scFv, which comprises humanized heavy- and light-chain variable regions fused with a (Gly_4_Ser)_3_ linker and cloned into the Fc-hole (H-Fc) expression vector. Heavy- and light-chain variable-region genes of the CD3-targeting antibody OKT3 were synthesized and fused with a (Gly_4_Ser)_3_ linker to yield the OKT3 scFv, which was then cloned into the Ig Fc-knob (K-Fc) expression vector.

### Expression of the anti-CD3/gB bispecific antibody.

Plasmids for OKT-scFv-K-Fc and Hu272.7-scFv-H-Fc were cotransfected at a ratio of 1:1 into HEK293F cells in the presence of branched polyethylenimine (Sigma, St. Louis, MO) as described previously (38, 39). The cell culture supernatant was harvested 7 days after transfection and was purified by protein A. The purified antibody was validated by SDS-PAGE under nonreducing conditions.

### ELISA.

ELISA were used to determine endpoint antibody binding titers to gB. The gB protein was immobilized at 2.0 μg/ml in phosphate-buffered saline (PBS) on 96-well Nunc-Immuno plates at 4°C overnight. Plates were blocked with 3% (vol/vol) nonfat milk in PBS–0.05% Tween 20 and were then incubated for 1.5 h with serial 3-fold antibody titrations starting at 10 μg/ml. Plates were washed after serum incubation. Then horseradish peroxidase (HRP)-conjugated goat anti-human or goat anti-rabbit IgG (Southern Biotech, Birmingham, AL) was added to the plates for 60 min, followed by another wash. A fluorogenic HRP substrate, 10-acetyl-3,7-dihroxyphenoxazine (ADHP) (Virolabs, Chantilly, VA), was then added for 3 to 5 min at 100 μl per well to generate resorufin at a concentration proportional to the HRP concentration. Fluorescent signals with excitation at 531 nm and emission at 595 nm were measured with a plate reader (Victor III; PerkinElmer). EC_50_ binding values were calculated from four-parameter curve fitting using Prism 5.

### Flow cytometry.

Purified human T cells were washed twice in PBS and suspended in staining buffer (PBS, 5% FBS, and 0.05% NaN_3_). Cells (1 × 10^6^) were stained for 1 h at 4°C with 1 μg/ml MAb hu272.7 or 1 μg/ml bispecific antibody. Cells were washed twice in PBS and were then incubated with 1 μg/ml biotinylated gB protein for 1 h at 4°C. After two washes with PBS, 100 μl of PE-conjugated streptavidin diluted at 1:5,000 was added to each sample. The cells were then incubated for 1 h at 4°C, washed twice with PBS, and analyzed by flow cytometry on a Guava easyCyte flow cytometer (Millipore, Hayward, CA). The cell populations of interest were gated and were analyzed using FlowJo software (FlowJo, Ashland, OR).

For flow cytometry analysis, the following reagents were used: fluorescein isothiocyanate (FITC)-conjugated anti-CD3D (clone S4.1; Invitrogen, Carlsbad, CA), PE-Cy5.5-conjugated anti-CD4 (OKT4; Stemcell Technologies, Vancouver, BC, Canada), PE-conjugated anti-CD8 (clone SK1; Biolegend, San Diego, CA), allophycocyanin (APC)-conjugated anti-CD25 (clone BC96; Biolegend, San Diego, CA), APC-conjugated anti-CD69 (clone FN50; Biolegend, San Diego, CA), and APC-conjugated anti-CD107a (clone H4A3; Biolegend, San Diego, CA).

### Detection of gB on the surfaces of HCMV-infected cells.

ARPE-19 cells were seeded in 24-well plates (1 × 10^5^ cells/well) and were cultured for 1 day. The cells were inoculated with AD169 revertant HCMV (MOI, 10) for 2 h at 37°C. The virus was then removed, and fresh medium with 2% FBS was added to the cells. The cells were detached from the plate using enzyme-free cell dissociation buffer (Gibco, Waltham, MA) at days 1, 2, 3, and 4 after infection and were subjected to gB staining. The cells were washed twice with cold PBS and were blocked with 3% BSA for 1 h on ice. The cells were stained with 10 μg/ml anti-gB (humanized antibody 272.7) for 1 h on ice, followed by staining with Cy3-conjugated anti-human F(ab′)_2_ (Jackson ImmunoResearch Laboratories, West Grove, PA) for 40 min on ice. The cells were washed twice with PBS after each staining. The gB on the cell surface was detected using a Guava easyCyte HT flow cytometer as described above.

### T cell activation and cytokine quantification assays.

ARPE-19 cells were infected with the AD169 revertant HCMV at an MOI of 5 for 72 h. Fresh T cells isolated from PBMCs were added to the infected cells at an E:T (immune effector-to-target cell) ratio of 20:1 with the bispecific antibody at different concentrations. Briefly, the bispecific antibody was 3-fold serially diluted starting at 1 μg/ml. Antibody hu272.7 was used as a negative control. T cells were incubated with 100 μl of the bispecific or hu272.7 antibody and an anti-CD28 antibody (2 μg/ml) at 37°C under 5% CO_2_ in a humidified incubator for 48 h. Cell culture supernatants were harvested and were utilized for cytokine quantification using a human TNF-α, IFN-γ Quantikine ELISA kit (R&D Systems, Minneapolis, MN). T cells were collected for the detection of the surface activation markers CD25, CD69, and CD107a by flow cytometry.

### Immunofluorescence microscopy.

The interaction between human T cells and HCMV-infected epithelial cells was analyzed by microscopy as described previously (40). ARPE-19 cells (1 × 10^4^/well) were seeded in 6-well plates for 24 h and were infected with AD169 revertant GFP-HCMV at an MOI of 5. At 48 h after infection, T cells (2 × 10^5^) isolated from fresh PBMCs and the bispecific antibody at a concentration of 1 μg/ml were added to the wells. Non-virus-infected ARPE-19 cells were used as a negative control. The colocalization of T cells and ARPE-19 cells with or without AD169 revertant GFP-HCMV infection was examined under microscopy 24 h later.

### Cytotoxicity assay.

Killing of HCMV-infected ARPE-19 cells was monitored continuously and noninvasively using the xCELLigence system (ACEA Bioscience, San Diego, CA) as described previously (41). The xCELLigence system of real-time cell analyzers allows for continuous, label-free, dynamic monitoring of cellular phenotypic changes by measurement of electrical impedance. The system measures impedance using interdigitated microelectrodes integrated into the bottom of each well of the tissue culture E-Plates 96. Impedance measurements are displayed as cell index (CI) values, providing quantitative information about the biological status of the cells, including cell number and cell viability. The result of impedance-based monitoring of cell viability correlates with that of the cell number- and 3-(4,5-dimethyl-2-thiazolyl)-2,5-diphenyl-2H-tetrazolium bromide (MTT)-based method (42). Briefly, 48 h after infection, HCMV-infected ARPE-19 cells were seeded into an E-plate 96 (ACEA Bioscience, San Diego, CA) as target cells (T), followed by the addition of T cells as immune effector cells (E), in the presence of the 3-fold serially diluted bispecific antibody. Cells alone were used as a baseline control. Three wells were assayed for each treatment group, where the average for the three wells was used as the cell growth index. Cell growth (measured as the cell index) was monitored continuously for 60 h. The E:T ratio was 20:1. The cell index was normalized when the effector cells were added. The normalized cell index, recorded 30 h after the addition of antibodies, was used to calculate the percentage of inhibition of the growth of HCMV-infected ARPE-19 cells using a formula described in other studies: [(cell index of the T cell group) − (cell index of the T-cell-plus-antibody group)/(cell index of the T cell group)] × 100% (43, 44). The results were expressed as means ± standard deviations (SD). Experiments were repeated three times, with three replicates for each treatment.

### Statistical analysis.

Unpaired two-tailed *t* tests or two-way analysis of variance (ANOVA) was performed where indicated and was analyzed using Prism 5 (GraphPad Software, La Jolla, CA). Statistical significance is indicated as follows: n.s., *P* > 0.05; **, *P* < 0.01; ***, *P* < 0.001. All results are presented as mean values ± SD.
